# The Nectin family ligands, PVRL2 and PVR, in cancer immunology and immunotherapy

**DOI:** 10.3389/fimmu.2024.1441730

**Published:** 2024-08-02

**Authors:** Kosuke Murakami, Sudipto Ganguly

**Affiliations:** The Bloomberg~Kimmel Institute for Cancer Immunotherapy, Johns Hopkins University School of Medicine, Baltimore, MD, United States

**Keywords:** cancer immunotherapy, DNAM1, immune checkpoint inhibitor, PVR, PVRIG, PVRL2, TIGIT

## Abstract

In recent years, immunotherapy has emerged as a crucial component of cancer treatment. However, its efficacy remains limited across various cancer types, highlighting unmet needs. Poliovirus receptor-related 2 (PVRL2) and Poliovirus receptor (PVR) are members of the Nectin and Nectin-like Molecules family, known for their role as cell-cell adhesion molecules. With the development of immunotherapy, their involvement in tumor immune mechanisms as immune checkpoint factors has garnered significant attention. PVRL2 and PVR are predominantly expressed on tumor cells and antigen-presenting cells, binding to PVRIG and TIGIT, respectively, which are primarily found on T and NK cells, thereby suppressing antitumor immunity. Notably, gynecological cancers such as ovarian and endometrial cancers exhibit high expression levels of PVRL2 and PVR, with similar trends observed in various other solid and hematologic tumors. Targeting these immune checkpoint pathways offers a promising therapeutic avenue, potentially in combination with existing treatments. However, the immunomodulatory mechanism involving these bindings, known as the DNAM-1 axis, is complex, underscoring the importance of understanding it for developing novel therapies. This article comprehensively reviews the immunomodulatory mechanisms centered on PVRL2 and PVR, elucidating their implications for various cancer types.

## 1 Introduction

In recent years, immunotherapy has become a new treatment modality for solid and non-solid cancers (1–3). It has become an essential pillar of cancer treatment, along with surgery, chemotherapy, and radiation therapy. Among immunotherapies, anti-CTLA4 and anti-PD-1/PD-L1 antibodies are the most common immune checkpoint inhibitors (ICIs) for solid tumors (3). Starting with the 2011 FDA approval of the anti-CTLA4 antibody, Ipilimumab, for metastatic melanoma (4), the anti-PD-1 antibodies (e.g., nivolumab, pembrolizumab, dostarlimab) and the anti-PD-L1 antibodies (e.g., atezolizumab, durvalumab, avelumab) have been FDA-approved for a variety of cancers (5). ICIs are a very promising treatment, sometimes affecting dramatically in cases that would have otherwise had no effective treatment (5). On the other hand, the therapeutic efficacy of existing ICIs is limited, and even in potentially effective carcinomas, response rates are limited to 20-40% (6). In addition, there are many cases in which acquired resistance develops during the treatment course (7, 8). Therefore, various attempts have been made to improve therapeutic efficacy, including developing new ICIs and combination therapy. Recently, combination therapy with a new ICI, anti-LAG3 antibody (relatlimab) and anti-PD-1 antibody (nivolumab) was shown to be effective in melanoma and was FDA-approved (9).

Recently, the Poliovirus receptor-related 2 (PVRL2)-Poliovirus receptor-related immunoglobulin (Ig) domain containing (PVRIG) pathway and the Poliovirus receptor (PVR)-T cell immunoreceptor with Ig and immunoreceptor tyrosine-based inhibitory motif (ITIM) domains (TIGIT) pathway have been focused on as potential new targets for cancer immunotherapy (10). These pathways are entirely different from the CTLA4 and PD-1/PD-L1 pathways, which have been previously targeted. The DNAX Accessory Molecule-1 (DNAM1) axis, including the PVRL2-PVRIG and PVR-TIGIT pathways, is a complex immunoregulatory mechanism, but recent studies have revealed much. This review article will focus on PVRL2 and PVR, which play vital roles in the DNAM1 axis, from the viewpoint of tumor immunity.

## 2 PVRL2 and PVR as the Nectin and Nectin-like molecule family

PVRL2, also known as Nectin-2, CD112, or PRR2, is a member of the Nectin family. PVR, also known as Necl-5, CD155, or TAGE4, has a domain structure like nectin and is one of the Nectin-like Molecules (Necls) (11–13). The Nectin and Necl Family have been identified to date as four types of Nectins (Nectin-1 to Nectin-4) and five types of Necls (Necl-1 to Necl-5), but they were discovered in different ways and given diverse names (11–13). In this article, the terminology is unified as PVRL2 and PVR.

Nectin and Necl family are immunoglobulin-like transmembrane cell adhesion molecules expressed in various cell types (14–16). Nectins are mainly involved in cell-cell adhesion, and Necls have a greater variety of cellular functions (14). They are involved in the organogenesis of sperm, eyes, inner ear, teeth, cerebral cortex, and nerves (12). Cell-cell adhesion can be involved in various diseases, among which the Nectin and Necl family is well known to be associated with Alzheimer's disease, mental disorders, viral infections, and cancer (12). In cancer, the expression of adhesion factors on the cell surface plays an essential role in dissemination, metastasis, and growth (17). The Nectin and Necl family also plays a vital role as a member of immunomodulatory mechanisms and is deeply involved in tumor progression (11).

**Figure 1:**
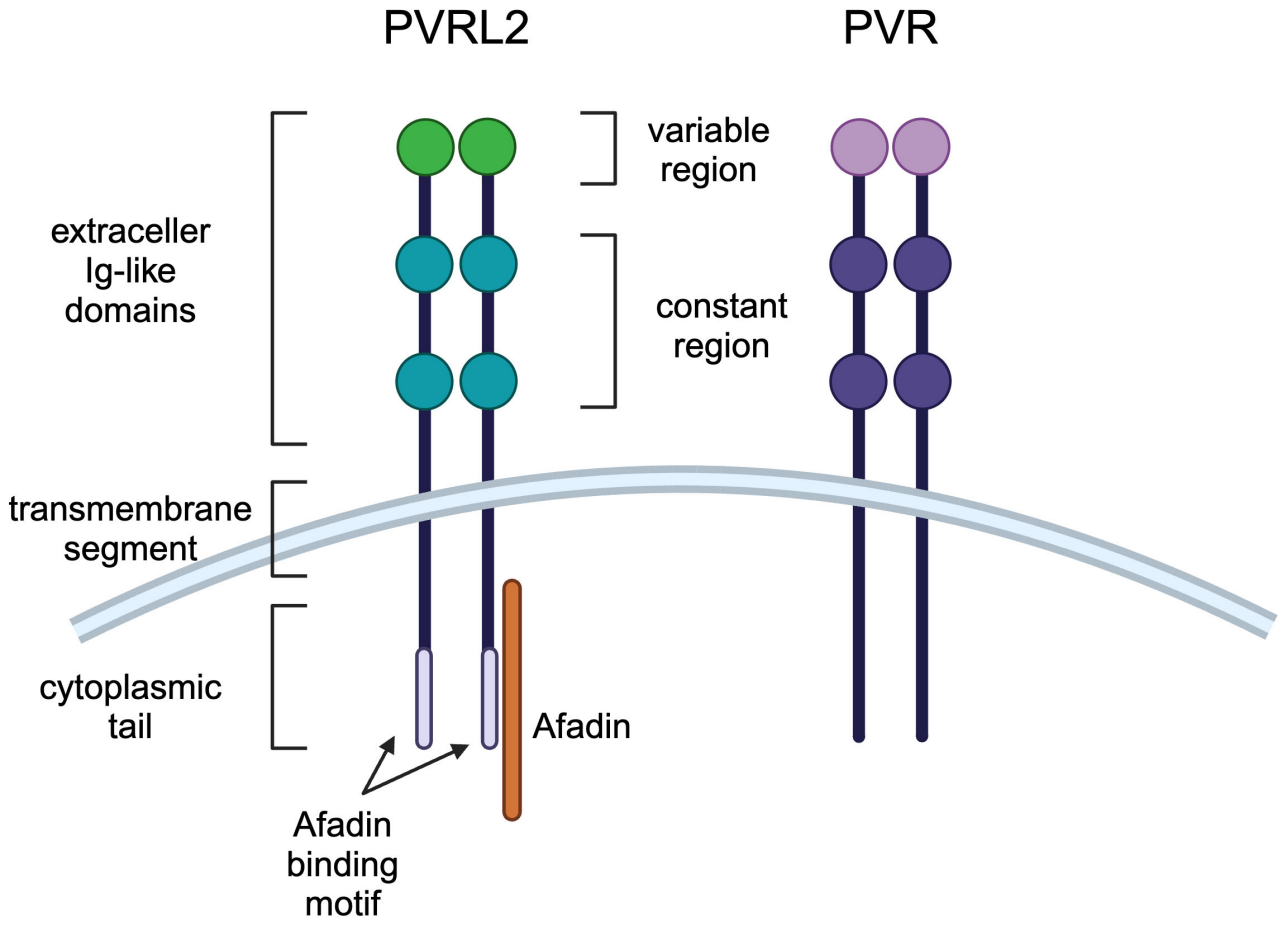
Molecular structures of PVRL2 and PVR. PVRL2 and PVR have the same structure of three extracellular Ig-like domains (one variable region and two constant regions) followed by a transmembrane region and a cytoplasmic tail. PVRL2 has an Afadin binding motif in the cytoplasmic tail, but PVR doesn't have it.

Cell-cell adhesion is mediated by tight junctions, adherens junctions, and desmosomes, and the Nectin and Necl family localizes to adherens junctions (16). Nectin has three extracellular Ig-like domains (one variable region and two constant regions) followed by a transmembrane region and a cytoplasmic tail (**Figure 1**) (15). The cytoplasmic tail of nectin other than nectin-4 contains a conserved Afadin binding motif, Glu/Ala-X-Tyr-Val, to which the PDZ domain of Afadin binds, causing nectin to bind to actin filaments (18). Necls have the same domain structure as nectin but do not have an Afadin binding motif in their cytoplasmic tail (**Figure 1**) (19). PVR most closely resembles nectin compared to other Necls, with PVRL2 and PVR having 51.7% amino acid sequence identity in the extracellular region (12, 20).

Cell adhesion molecules form trans dimers and mediate cell-cell adhesion by trans interaction (12). Cadherins form only trans homo dimers, whereas the Nectin and Necl families form not only trans homo dimers but also trans hetero dimers between family members (14, 19, 21). PVRL2 has homophilic and heterophilic and PVR has heterophilic cell-cell adhesion activity (12, 21). Unlike cadherins, the Nectin and Necl family is also expressed in immune cells such as T and NK cells and is characterized by its involvement in immune mechanisms (22).

## 3 The function of the PVRL2 and PVR in the immune system

**Figure 2:**
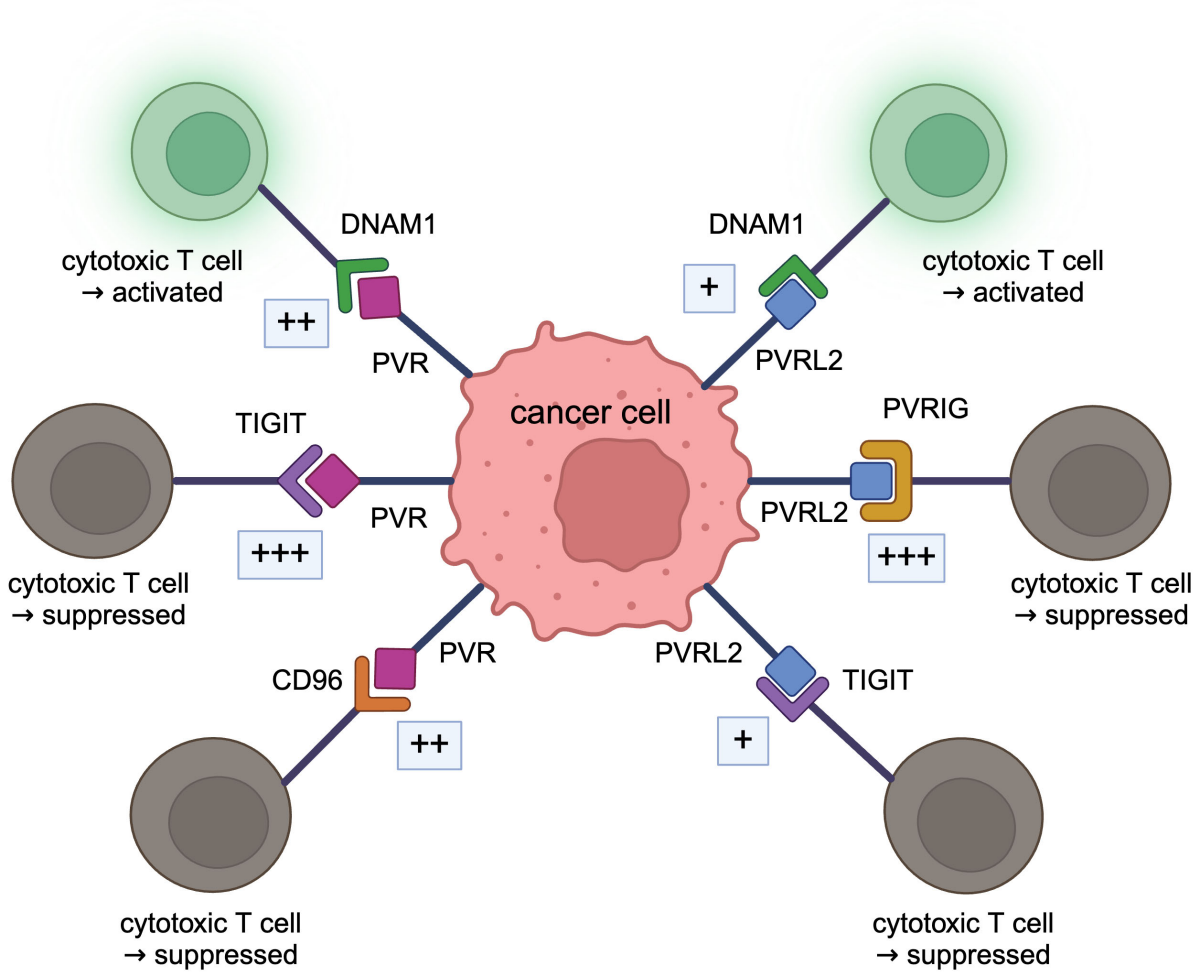
DNAM1 axis. PVRL2 binds to PVRIG and TIGIT, and PVR binds to TIGIT and CD96, acting in a tumor immunosuppressive manner. These bindings are stronger than the immunostimulatory bindings of DNAM1 to PVRL2 or PVR.

While PVRL2 and PVR are known to be expressed on many tumor cells, they are also expressed on immune cells and widely regulate cell-cell interactions (22). However, most reports on PVRL2 and PVR in the immune system focus on the interaction of PVRL2 and PVR expressed in tumor cells with T and NK cells. There is a lack of reports on how T and NK cells interact with PVRL2 and PVR expressed in immune cells, especially myeloid cells. In this section, we mainly summarize the functions of PVRL2 and PVR expressed on tumor cells, focusing on their immune system regulation (**Figure 2**).

### 3.1 PVRL2 (Nectin-2, CD112)

PVRL2 was reported as Poliovirus Receptor Related 2 (PRR2), a cell surface molecule homologous to PVR (15). The gene encoding this molecule has two mRNA isoforms, a 3.0 kb short form and a 4.4 kb long form (15). PVRL2 is ubiquitously expressed in various cells, including epithelial cells, endothelial cells, smooth muscle cells, and fibroblasts (23, 24). However, PVRL2 expression in immune cells is specific to myeloid cells, with little expression in lymphocytes (15). PVRL2 is also characterized by high expression in Sertori and Leydig cells in the testis and is involved in spermatogenesis (25). Disruption of PVRL2 results in male infertility (25). PVRL2 is also expressed in vascular endothelial cells and regulates angiogenesis (23, 24).

In the tumor immune axis, PVRL2 is expressed on tumor cells and tumor-infiltrating immune cells, especially macrophages, and is involved in tumor growth and metastasis (12, 13). For example, PVRL2 is highly expressed in CD14^+^ cells (as tumor-associated macrophages) and CD45^-^ cells (as tumor cells) in breast, endometrial, ovarian, lung, and liver cancers (26). Regarding regulation of expression on tumor cells, PVRL2 is primarily expressed in the cytoplasm in tumor cell lines, and inhibition of the ubiquitin pathway has been shown to increase surface expression of PVRL2 and increase tumor cell sensitivity to NK cell cytotoxicity (27).

### 3.2 PVR (Necl-5, CD155)

In 1989, it was reported that human PVR is a transmembrane protein with an amino acid and domain structure like nectins, which is characteristic of the Ig superfamily and is expressed in various human tissues (28). Like PVRL2, PVR is expressed on epithelial cells, endothelial cells, smooth muscle cells, and fibroblasts, as well as on immunocompetent cells such as Neutrophils, T cells, and plasma cells (29). Quantification of PVR mRNA in normal human tissues shows the highest expression in the liver (30). Soluble PVR isoforms are present in human serum and cerebrospinal fluid (30, 31).

PVR is involved in cell migration; PVR has been shown to interact with the platelet-derived growth factor (PDGF) receptor to regulate the interaction between the PDGF receptor and integrin, effectively inducing directional cell migration (32). PVR also attracts growing microtubules to the plasma membrane of the leading edge of moving cells (33). PVR is also involved in cell proliferation; PVR has been shown to promote activation of Ras-Raf-MEK-ERK signaling, increase cyclin D2 and E expression, decrease p27Kip1, and shorten the G0/G1 phase of the cell cycle (34). It has also been suggested that PVR may regulate angiogenesis by vascular endothelial growth factor (VEGF) (35). In the immune system, PVR is involved in negative selection in the thymus using a PVR-deficient mouse model (36). A pathway dependent on the DNA damage response is involved in the elevated expression of PVR (37). It has also been shown that DNA damage downregulates PVR expression (38, 39). Hedgehog signaling is often activated in cancer (40), and PVR is upregulated by the signaling (41). PVR expression is induced through the Raf-MEK-ERK-AP-1 pathway by upregulating fibroblast growth factor or KRAS (42).

### 3.3 Receptor expression and regulatory mechanisms

As receptors for PVRL2 in the immune system, Poliovirus receptor-related immunoglobulin domain containing (PVRIG, CD112R), T cell immunoreceptor with Ig and ITIM domains (TIGIT, VSTM3, VSIG9, WUCAM), DNAX Accessory Molecule-1 (DNAM1, CD226, TLiSA1) are known. The binding of PVRL2 to PVRIG or PVRL2 to TIGIT suppresses tumor immunity, whereas the binding of PVRL2 to DNAM1 promotes tumor immunity (10). TIGIT, DNAM1, and CD96 (TACTILE) are known receptors for PVR in the immune system; binding of PVR to TIGIT suppresses tumor immunity while binding to DNAM1 promotes tumor immunity; binding to CD96 acts both to suppress and promote tumor immunity (10).

PVRIG is a transmembrane protein consisting of an extracellular IgV domain, a transmembrane domain, and an intracellular ITIM-like motif with a high affinity for PVRL2 (43). PVRIG is expressed in human T and NK cells and functions as an immune checkpoint pathway (43). PVRIG is not expressed in dendritic cells, neutrophils, monocytes, or B cells (43). The expression of PVRIG in T cells can vary with the expression of other immune checkpoint factors. For example, PVRIG has been shown to correlate with the expression of T cell exhaustion markers such as PD-1 and TIGIT in CD8^+^ T cells and CD4^+^ T cells (26). It has also been shown that the blockade of PVRIG increases TIGIT expression, but the blockade of TIGIT or PD-1 does not alter PVRIG expression (26).

TIGIT is a member of the Ig superfamily and is a transmembrane protein that contains an extracellular IgV domain, a transmembrane domain, and a cytoplasmic tail containing an ITIM and an Ig tail tyrosine (ITT)-like motif (44). TIGIT is expressed on various T cell subsets, NK, and NKT cells in humans (44–50). TIGIT is a receptor for PVRL2 and PVR (44, 51). TIGIT is the most crucial receptor for PVR; compared to the binding of PVRL2 and TIGIT, the binding of PVR to TIGIT is very strong and strongly immunosuppressive (44). The TIGIT/PVR pathway suppresses IFN-γ production in NK cells (52). TIGIT is highly expressed predominantly in tumor-infiltrating T cells and plays a vital role in suppressing the activity of CD8^+^ T cells (53). TIGIT inhibits T cell cytotoxic function by competing with DNAM1, as discussed below (54). The binding of PVRL2 to TIGIT is much weaker than that of PVR to TIGIT, suggesting that it does not play a significant role in the tumor immune system.

CD96, another receptor for PVR, was reported in 1992 as a member of the Ig superfamily that activates T cells (55). CD96 is mainly expressed in T and NK cells (55, 56) and highly expressed in tumor-infiltrating CD8^+^ T cells (56, 57). It was also reported in 2014 that CD96 competes with and directly inhibits the binding of DNAM1 and PVR, thereby limiting NK cell function (58). CD96 is also expressed on tumor cells and is associated with chemotherapy resistance and poor prognosis (59).

DNAM1 is a member of the Ig superfamily of transmembrane proteins with two extracellular IgV domains and an intracellular ITIM-like motif (60). It is expressed in T, NK, B, and monocytes (60–64). It binds to both PVRL2 and PVR and promotes activation of T, NK, B, and monocytes (60–64). The expression of PVR enhances NK cell activity via the DNAM1 pathway (65). DNAM1, unlike PVRIG and TIGIT, exhibits antitumor activity by enhancing the cytotoxic activity of immune cells through ligand binding (66, 67). On the other hand, it has recently been reported that under inflammatory conditions, DNAM1 promotes IFN-γ secretion by conventional CD4^+^ T cells and contributes to tumorigenesis (68). The interaction between PVRL2 and DNAM1 requires the homodimerization or engagement of the homodimeric interface of PVRL2 IgV (22). DNAM1 has a soluble form and can bind to PVRL2 or PVR on tumor cells (69).

### 3.4 Relationships of PVRL2 and PVR in the DNAM1 axis

DNAM1 axis, including PVRL2 and PVR, is very complex (10), and the relationship needs to be well organized. PVRL2 and PVR are both ligands for DNAM1 (62, 63). Both PVRL2 and PVR, which are expressed on antigen-presenting cells, act in an immunostimulatory manner when bound to DNAM1 to activate T and NK cells (70), but the binding between PVR and DNAM1 is stronger than that between PVRL2 and DNAM1 (63). PVRL2 binds to PVRIG and is expressed on T and NK cells, acting immunosuppressively. Interestingly, the immunosuppressive binding of PVRL2 to PVRIG is stronger than the immunostimulatory binding of PVRL2 to DNAM1 (43). PVRIG binds specifically to PVRL2, and there are no reports of PVRIG acting with PVR. Both PVRL2 and PVR also bind to TIGIT and inhibit the cytotoxic activity of immune cells (46). The action of PVRL2 and TIGIT is the same as that of PVR and TIGIT (71). In the past, TIGIT expressed on human NK cells was shown to bind to PVR and PVRL2 and inhibit tumor killing by NK cells (46). Recently, however, it has been demonstrated that the binding of PVRL2 to TIGIT is very weak, with little interaction (26, 43, 51). In mice, binding to TIGIT is restricted to PVR (72). The binding of PVR to TIGIT is very strong and exhibits strong immunosuppressive activity (44). The binding of PVR to TIGIT is stronger than PVR to DNAM1 (44, 73). PVR also binds to CD96, expressed in T and NK cells (55). This binding has been shown to stimulate the cytotoxicity of activated NK cells and act in an immunostimulatory manner (74), while anti-CD96 antibodies stimulate the cytotoxic function of NK cells (75). Signaling through CD96 has also been shown to enhance the cytotoxic activity of CD8^+^ effector T cells (76). Therefore, whether this pathway acts in an immunostimulatory or inhibitory direction is not constant. The affinity between CD96 and PVR is higher than that between PVR and DNAM1 but lower than between PVR and TIGIT (43, 44).

Blockade of each pathway of the DNAM1 axis has been shown to affect the regulation of immune mechanisms. In PVRIG-deficient mouse models of melanoma and colorectal cancer, tumor growth is suppressed, and tumor-infiltrating CD8^+^ T cells are increased (77). PVRIG inhibition effectively suppresses tumor growth and prolongs the survival of tumor-bearing mice, which is associated with enhanced frequency and cytotoxicity of tumor-infiltrating NK cells (78). TIGIT inhibition also activates T cells (79). Although blockade of either PVRIG or TIGIT slightly increases mitosis and cytokine production in CD4^+^ T cells (43), double block of PVRIG and TIGIT has been shown to enhance significantly CD4^+^ T cell proliferation and cytokine secretion, including IFN-γ, IL-13, IL-10, IL-5, IL-13, IL-10, IL-5, IL-2, and enhances the cytotoxicity of CD8^+^ T cells (26, 43). It also reduces the cytotoxicity of T cells and NK cells by blocking DNAM1 (62, 63, 73). It has also been shown that increased CD8^+^ T cells with high DNAM1 expression improve response to anti-TIGIT therapy (80).

## 4 PVRL2 and PVR in cancer

So far, we have discussed the complex expression of PVRL2 and PVR and their immunomodulatory mechanisms. In this section, we summarize the expression of PVRL2 and PVR in cancer and their effects on tumor immunoregulatory mechanisms (**Table 1**).

**Table 1:** PVRL2 and PVR in various cancers.

	PVRL2 expression	tumor growth prognostic effect	PVR expression	tumor growth prognostic effect	evidence for treatment
Solid tumor					
ovarian	●	pro-tumor	●	pro-tumor	anti-TIGIT, anti-PVRIG
endometrial	●		●	pro-tumor	
liver	●	pro/anti-tumor			
pancreas	●	pro-tumor	●	pro-tumor	anti-TIGIT
gastric	●	pro-tumor	●	pro-tumor	anti-TIGIT
colorectal	●	pro-tumor	●	pro-tumor	anti-PVRIG
breast	●	pro-tumor	●	pro-tumor	anti-TIGIT
gallbladder	●	pro-tumor	●	pro-tumor	
melanoma			●	pro-tumor	anti-TIGIT
prostate			●	pro-tumor	anti-TIGIT
head and neck			●	pro-tumor	
esophageal			●	pro-tumor	
lung			●	pro-tumor	
bladder			●	pro-tumor	
cervical			●	pro-tumor	
Hematologic tumor					
leukemia	●	pro-tumor	●	pro-tumor	
lymphoma			●	pro-tumor	

● expression (+).

In 2013, gene expression profiling and immunohistochemistry (IHC) showed that PVRL2 is overexpressed in breast and ovarian cancer clinical tissues (81). Subsequently, The Cancer Genome Atlas Program has published mRNA expression data for several cancers and found high expression of PVRL2 mRNA in breast, ovarian, prostate, endometrial, gastric, liver, pancreatic, and lung cancers, with no carcinoma specificity (26). Expression of PVRL2 is found in both PD-L1 negative and PD-L1 positive tumors (26).

PVR is overexpressed in many cancers compared to normal tissue and has been shown to correlate with poor prognosis (82, 83). It has also been demonstrated that PVR expression correlates with poor prognosis in many cancers (82). Even if there is no prognostic difference by PD-L1 expression, there may be a prognostic difference by PVR expression (84). It has also been shown that patients with lung, gastrointestinal, breast, and gynecological cancers have higher levels of soluble PVR in their serum than healthy donors (31).

Depending on the type of cancer, either PVRL2 or PVR may be expressed predominantly. Breast, ovarian, endometrial, and prostate cancers express PVRL2 predominantly, while melanoma, esophageal, and colorectal cancers express PVR predominantly (26). There are also many reports on ligands for PVRL2 and PVR in cancer. PVRIG, the ligand for PVRL2, is upregulated in various carcinomas, including kidney, ovary, lung, prostate, and endometrial cancer (26). As mentioned above, TIGIT functions particularly strongly as a ligand for PVR and is highly expressed in tumor-infiltrating lymphocytes in many cancers, including endometrial, breast, renal cell, non-small cell lung, and colon cancer (53). It has also been shown that TIGIT^+^ CD8^+^ T cells frequently co-express PD-1 in melanoma and ovarian high-grade serous carcinomas (82, 85).

Heterogeneity of expression of immune checkpoint pathways in tumors is important for considering tumor immunity, but there are still very few reports on the heterogeneity of DNAM1 axis expression so far. Only one report has shown that the expression of PVR, PVRL2, TIGIT, PD-1, and PD-L1 in lung adenocarcinoma is heterogeneous within and between tumors and varies according to tumor growth patterns (86), so future reports are expected. In addition, even though PVRL2 and PVR are known to be expressed on both tumor cells and antigen-presenting cells (especially macrophages), most studies have focused on the expression on tumor cells.

Another critical factor in cancer is the change in expression that occurs with treatment, such as chemotherapy. *In vitro*, Adriamycin treatment of breast cancer cell lines increases PVR expression (39). The combination of PVR knockdown and Adriamycin administration induces more cell death and inhibits tumor growth than either one (39). Myeloma cells treated with doxorubicin, melphalan, and bortezomib also upregulate PVRL2 and PVR expression and suppress the antitumor effects of NK cells (87). Thus, it is suggested that chemotherapy causes changes in the expression of PVR and PVRL2, but there are still only a few reports. However, these effects must be considered in clinical practice.

## 5 What is known in detail about each cancer type?

### 5.1 Gynecologic Müllerian cancer

As mentioned above, PVRL2 and PVR are highly expressed in ovarian and endometrial cancers. Therefore, the significance of PVRL2 and PVR in these gynecologic Müllerian cancers is very important.

#### 5.1.1 Ovarian cancer

Ovarian cancer is one of the poorest prognoses among gynecologic cancers (88). Among epithelial ovarian cancer, about three-quarters are high-grade serous ovarian cancer (HGSOC) (89). HGSOC have a modest immunogenic repertoire (90). However, the tumor immune microenvironment is known to be highly suppressive (91). In the phase 3 clinical trials, ovarian cancer has a limited response to existing ICIs (92–96), and new therapeutic targets and combination therapies are expected to be developed.

In 2007, it was shown that PVR is expressed in ovarian cancer cells and enhances NK cell activity via the DNAM1 pathway (97). In clinical samples, PVR was also expressed well in tumor epithelial cells of HGSOC (82). In this report, PVR expression did not correlate with tumor-infiltrating lymphocytes, whereas PD-L1 was highly expressed on tumor-associated macrophages and positively correlated with TILs, indicating that PVR and PD-L1 have different expression patterns (82).

Recently, focal adhesion kinase (FAK) was reported to function in tumor immunosuppression of ovarian cancer via the PVR/TIGIT pathway (98). In this report, it was shown that FAK and PVR are coexpressed in tumor cells in HGSOC and that the combination of FAK inhibitors and anti-TIGIT antibodies maintains elevated TIL levels, decreases TIGIT-positive Tregs, prolongs survival, increases CXCL13, and leads to the formation of tertiary lymphoid structure in the ovary (98).

In 2013, gene expression profiling and immunohistochemical studies first demonstrated that PVRL2 is overexpressed in clinical ovarian cancer specimens and various human ovarian cancer cell lines (81). In another cohort, PVRL2 was identified as the highly expressed transcript, and immunohistochemistry confirmed that it is overexpressed in HGSOC samples compared to normal or benign samples (99). Similar results were also confirmed by analysis of gene expression data from TCGA (99). Bekes et al. reported that in a cohort of 60 patients with ovarian cancer, PVRL2 expression of patients with lymph node metastases or residual tumors after surgery had higher levels of PVRL2 gene expression than those with negative lymph nodes or complete tumor resection (100).

Regarding the tumor microenvironment, peritoneal biopsies showed clear co-localization of PVRL2 and CD31 in the vasculature, and PVRL2 expression was suppressed in the peritoneal endothelium of patients with high blood VEGF levels (100). *In vitro*, it has been reported that VEGF causes downregulation of PVRL2 and that PVRL2 knockdown in endothelial cells is associated with increased endothelial permeability (100). HGSOC often results in ascites accumulation, and PVRL2 may be a contributing factor.

However, the role of the DNAM1 axis in ovarian cancer through tumor immune mechanisms is still poorly understood. In addition, previous studies have focused on HGSOC. The frequency of endometriosis-associated ovarian cancers (EAOC), including clear cell carcinoma, is high in Japan and other Asian countries (101). The mechanism of occurrence of EAOC is entirely different from that of HGSOC (102). Clinical trials have suggested the efficacy of ICI for clear cell carcinoma (103, 104), and studies involving the DNAM1 axis are also expected.

#### 5.1.2 Endometrial cancer

Endometrial cancer is the fourth most common cancer in women and the most common gynecologic cancer in the United States (105). Endometrial cancer is an important disease because, unlike many other cancers, the number of patients is increasing (106). Hypermutators due to *POLE* mutations are present in about 10% of cases of endometrial cancer (107). In endometrial cancer, the frequency of DNA mismatch repair deficiency or microsatellite instability-high is about 30%, the highest among all cancer types (108). These endometrial cancers have high neoantigen loads and many TILs and are highly immunogenic (109). The efficacy of anti-PD-1 antibodies against endometrial cancer has also been shown in a clinical trial (110), suggesting that endometrial cancer and immunotherapy are compatible.

Analysis using TCGA data shows that both PVRL2 and PVR are highly expressed in endometrial cancer, but PVRL2 expression is more predominant (26). PVR^+^PVRL2^+^ tumor cells are the most abundant compared to other cancers and have higher PVRIG expression (26). It has also been reported that cancer-associated fibroblasts derived from endometrial cancer have decreased PVR expression and more substantial suppression of the cytotoxic activity of NK cells (111).

However, even though the DNAM1 axis seems to be a promising therapeutic target for endometrial cancer, no detailed studies have been reported other than these, and future development is expected.

### 5.2 Other solid tumors

#### 5.2.1 Liver cancer

Hepatocellular carcinoma (HCC) has a moderate tumor mutational burden (90). In the tumor immune microenvironment of HCC, PD-1 expression is upregulated in lymphocytes, and PD-L1 and PD-L2 expression is also upregulated in Kupffer cells and hepatic sinusoidal endothelial cells (112). Inflammatory response with PD-1 and PD-L1 overexpression is seen in 25% of HCC samples (112).

It has been shown by bulk and single-cell RNA sequencing and IHC that PVRL2 is overexpressed in HBV-associated hepatocellular carcinoma (113). A liver cancer model using PVRL2 knockout mice has been shown to restore T cell infiltration into tumors and reduce T cell exhaustion, suppressing tumor growth (113). On the other hand, a study of 159 human subjects diagnosed with hepatocellular carcinoma showed that PVRL2 expression in tumor specimens was lower than that in peritumoral liver tissue, and low PVRL2 expression was associated with poor prognosis (114), so the significance is controversial. Single-cell RNA seq analysis of hepatocellular carcinoma also suggests that blockade of the PVR/PVRL2 and TIGIT pathways may exert an antitumor effect (115).

#### 5.2.2 Pancreas cancer

Pancreatic ductal carcinoma (PDA) is one of the tumors with extremely low immunogenicity (90). Despite promising Phase 1 clinical trials, subsequent trials have not shown the efficacy of existing anti-PD-1/PD-L1 or anti-CTLA4 antibodies, except in mismatch repair deficient cases (116). Thus, immunotherapy for PDA remains challenging.

PVRL2 expression is found in about half of cases of pancreatic cancer but not in benign lesions or normal pancreatic tissue (117). PVR expression is also higher in the tumor than adjacent normal tissue (118). PVRL2 and PVR expression have been reported to be poor prognostic factors (117, 119, 120). It has also been reported that PVRL2 expression correlates not with prognosis but with histologic grade (121).

Regarding tumor immunity, PVR expression was inversely correlated with tumor-infiltrating lymphocytes (119). The percentage of DNAM1^+^ and CD96^+^ NK cells is significantly lower in pancreatic cancer patients than in healthy controls, and reduced percentages of DNAM1^+^ and CD96^+^ NK cells are associated with tumor histologic grade and lymph node metastasis (118). In a pancreatic cancer pre-clinical model, combining a neoantigen vaccine and anti-TIGIT/anti-PD-1 antibody enhances anti-tumor effects (122). A detailed analysis of human samples showed that pancreatic cancer has many TRM cells with a PD-1^+^TIGIT^+^ phenotype, and the promising potential of double blockade of PD-1 and TIGIT has been suggested (123).

#### 5.2.3 Gastric cancer

Esophagogastric adenocarcinoma (EGAC) is considered to have a moderate tumor mutational burden (90). Immunotherapy for EGAC is promising. Phase 3 clinical trials have shown the efficacy of anti-PD-1 antibodies, which are already FDA-approved (124).

TIGIT-positive and PD-1-positive tumor-infiltrating CD8-positive T cells are increased in gastric cancer (125, 126). In addition, gastric cancer tissues and cell lines overexpress PVRL2 and PVR (125, 126). Soluble PVR is significantly higher in gastric cancer patients and is reduced by surgical resection (30). High PVR, PVRL2, and TIGIT expression are associated with poor prognosis (126, 127). An immune checkpoint score system, including PVRL2 and PVR, can predict prognosis (128).

*In vivo*, PVR binds to TIGIT and inactivates CD8^+^ T cells, and targeting PVR-TIGIT promotes CD8^+^ T cell responses and improves survival in cancer-bearing mice (125). Furthermore, inhibition of both TIGIT and PD-1 further promotes CD8^+^ T cell activation and improves survival in tumor-bearing mice (125).

#### 5.2.4 Colorectal cancer

Among colorectal cancers, mismatch repair deficient or microsatellite instability-high populations are highly immunogenic, and ICI is effective (129). However, for mismatch repair proficiency or microsatellite stability, existing ICIs are poorly effective, and new strategies are required (129).

PVR is highly expressed in patients with colorectal cancer (130). In 100 patients with stage III colorectal cancer, high expression of PVR and TIGIT were independent poor prognostic factors (131). Serum PVRL2 levels in patients with colorectal cancer were significantly higher than those in healthy controls (132). Expression of DNAM1 on CD8 T cells infiltrating liver metastases has also been shown to be a favorable prognostic factor (133).

In a colorectal cancer model, PD-L1 blockade inhibited tumor growth in PVRIG-deficient mice but not in wild-type mice (77). Furthermore, anti-PVRIG antibodies have been reported to inhibit tumor progression alone or in combination with PD-L1 inhibitors (77). These data are valuable because they demonstrate the efficacy of existing ICI and anti-PVRIG antibody combination therapy.

#### 5.2.5 Breast cancer

Triple-negative breast cancer (TNBC), which accounts for 15-20% of all breast cancers, is one of the most promising cancers for immunotherapy (134). In the past, chemotherapy was the only option, but now, many anti-PD-1/PD-L1 and anti-CTLA4 antibodies are FDA-approved and used in clinical practice (134). However, their efficacy is still far from satisfactory, and there is a need for further development of immunotherapy.

In breast cancer patients, high PVR expression is associated with poor prognosis (135–137). In examining PVR expression and NK cell infiltration by IHC, PVR expressed on the plasma membrane strongly correlates with the tumor-infiltrating NK cells (138). PVR expression is also associated with a mesenchymal phenotype (135). PVR secreted by Brain metastasis cancer-associated fibroblasts has been shown to enhance the invasive potential of cancer cells (139). Serum-soluble PVR levels correlate with risk factors for breast cancer (140). In addition, TIGIT expression correlates with age and histologic grade in triple-negative breast cancer (141) and correlates with poor prognosis in invasive breast cancer (142). Single-cell RNA sequencing analysis suggests that the PVRL2-TIGIT pathway promotes immune escape and lymph node metastasis (143, 144).

*In vitro* analysis showed that blockade of PVRIG or TIGIT increased the number of IFN-γ-producing NK cells under co-culture of breast cancer cells, and the combination of anti-PVRIG and anti-TIGIT antibodies induced a further increase in IFN-γ-producing NK cells and improved cytotoxicity of NK cells (145). Although the direct relationship to immune mechanisms is unclear, the knockdown of PVR also induced mesenchymal-epithelial transition of TNBC cells, inhibited TNBC cell migration, invasion, and metastasis *in vitro* and *in vivo*, and inhibited TNBC cell growth and survival (135).

#### 5.2.6 Melanoma

Melanoma is one of the most immunogenic tumors (90). Indeed, the development of immunotherapy has revolutionized the treatment of melanoma (146). Nevertheless, there is a need for different therapeutic strategies, as many cases do not respond to existing ICIs or become resistant to treatment at an early phase (146).

PVR is overexpressed in melanomas compared to melanocytes and benign nevi and correlates with known poor prognostic factors (147). Tumors with high PVR expression before treatment have a higher proportion of PD-1^+^ CD8^+^ T cells, which correlates with a lower response to anti-PD-1 and anti-PD-1/CTLA4 combination therapy (148).

PVR is a vital ligand recognized by DNAM1 in inhibiting NK cell-mediated melanoma metastasis (149). *In vivo*, soluble PVR inhibits DNAM1-mediated NK cell cytotoxic activity and promotes melanoma lung metastasis (150). TIGIT has also been well studied in melanoma and has been shown to regulate cytotoxic responses of T cells via the TIGIT-PVR pathway (85, 151). Analysis of tumor cell lines and paired TILs from ICI-treated melanoma patients showed high expression of PVR, TIGIT ligand, and TIGIT in tumor cell lines and tumor-infiltrating T cells, respectively, and functional assays showed that TIGIT blockade or PVR deletion activated T cells (10.1136/jitc-2021-003134). PVR expression was increased in surviving tumor cells after co-culture with TILs from the responder and inhibited TIGIT^+^ T cell activation (152). The combination of IL15 and TIGIT blockade has also been shown to restore the cytotoxicity of NK cells mediated by PVR (153).

#### 5.2.7 Prostate cancer

Prostate cancer has low immunogenicity and a suppressive tumor immune microenvironment (154). Therefore, anti-PD-1/PD-L1 and anti-CTLA4 antibodies have minimal efficacy (154).

In castration-resistant prostate cancer, blocking the TIGIT/PVR pathway with an anti-TIGIT monoclonal antibody has been shown to enhance the antitumor effect of NK cells (155). In addition, there are reports on the expression of PVR and PVRL2 in many other cancers. The expression of PVR has been reported as a poor prognostic factor in head and neck cancer (156), esophageal cancer (157), lung cancer (84, 158–160), and bladder cancer (161, 162). In cervical cancer, PVR expression increases as cervical lesions progress (163). It has also been reported that miRNAs regulate PVR expression in lung adenocarcinomas (164). It has also been shown that PVRL2 expression is a poor prognostic factor in gallbladder cancer (165) or glial tumors of the brain (166). PVRL2 is highly expressed in esophageal squamous cell carcinoma and is associated with advanced-stage and histologic differentiation (167). It has also been shown that decreased PVR expression induces cell apoptosis and inhibits tumor cell growth by inhibiting PI3K/Akt and MAPK signaling pathways (168). However, none of these reports have directly demonstrated a role for PVRL2 or PVR in tumor immune mechanisms.

### 5.3 Hematologic tumor

#### 5.3.1 Acute myeloid leukemia

TIGIT, PVR, and PVRL2 are highly expressed in AML patients, and high expression of PVR and PVRL2 correlates with poor prognosis (169–171). *In vitro*, inhibition of PVR2 or PVR with antibodies has a higher therapeutic effect on AML cell lines (170). Knockdown of TIGIT also restores CD8^+^ T cell dysfunction (169).

There are reports that PVRIG is expressed in NK cells of AML patients (172) and that DNAM1 is highly expressed in AML and is a prognostic factor (173).

#### 5.3.2 Lymphoma

In cutaneous T-cell lymphoma (CTCL), PVR is highly expressed in tumor cells (174). It is interesting to note that the expression of DNAM1 on NK cells and CD8^+^ cells in the peripheral blood of CTCL patients was decreased, while DNAM1 levels in the serum were increased, strongly reflecting disease activity, suggesting that soluble DNAM1 in the serum was generated by the shedding of membrane-form DNAM1 (174).

## 6 Therapeutic applications and clinical trials

The front runner in therapies targeting the DNAM1 axis is an anti-TIGIT antibody. Several mechanisms have been proposed for the immunosuppressive mechanism of TIGIT: cell-extrinsic mechanisms include the interaction of TIGIT with PVR, which has been shown to regulate cytokine production by DCs and affect T cell activity (44). Cell-intrinsic mechanisms are also thought to be involved, and agonistic anti-TIGIT mAbs have been shown to inhibit human and mouse T cell proliferation and cytokine production via anti-CD3/anti-CD28 mAbs in the absence of antigen-presenting cells (47). Thus, TIGIT blockade may inhibit these mechanisms and activate T cell cytotoxicity. Since the introduction of anti-TIGIT antibodies in 2018 (175), many clinical trials are ongoing (10). TIGIT and PD-1 can be co-expressed in tumor-infiltrating lymphocytes, and inhibition of both checkpoint pathways leads to greater activation of CD8^+^ T cell effector functions (53). Therefore, the efficacy of combination therapy with anti-TIGIT and anti-PD-1/PD-L1 antibodies is also very promising. In non-small cell lung cancer, a phase II trial of anti-TIGIT antibody (tiragorumab) plus anti-PD-L1 antibody (atezolizumab) demonstrated an objective response rate of 31.3% vs. 16.2%, compared with placebo plus atezolizumab. Median progression-free survival was 5.4 months (95% CI: 4.2-not estimable) vs. 3.6 months (2.7-4.4) for placebo plus atezolizumab (176). Several phase III clinical trials are currently underway for anti-TIGIT antibodies. Although in the preclinical stage, the usefulness of TIGIT and PD-1 chimeric immune-checkpoint switch receptors has also been reported (177).

A highly reactive anti-PVRIG drug known as COM701 is in its first clinical trial phase (178). The combination of COM701 and COM902, an anti-TIGIT monoclonal antibody, has shown good antitumor effects *in vivo* and is expected to have clinical applications (179).

A clinical trial of DNAM1 agonist for solid tumors was also underway, but this study was terminated due to a strategic business decision made by the company (180).

The anti-PVRL2 monoclonal antibody has also been shown to exert anti-tumor effects by antibody-dependent cellular cytotoxicity *in vitro* or *in vivo* (81). Antibody-drug conjugates targeting PVRL2 therapeutically have also been shown to exert antitumor effects in a mouse xenograft model (181), and Fc2-modified anti-PVRL2 antibodies have been shown to exert antitumor effects with controlled adverse effects in monkeys (182). There is also a report that Bispecific anti-CD3 x anti-CD155 antibody is effective against hematologic cancers (183), which is an interesting new direction.

Clinical trials of PVSRIPO in patients with recurrent malignant glioblastomas (184) and unresectable melanoma treated with intratumoral PVSRIPO (185) have all shown promising results. Still, these are direct effects on the tumor cells.

## 7 Conclusions

Among the Nectin and Necl family members, PVRL2 and PVR are critical players in tumor immunity, and immune checkpoint pathways mediated by them are potential therapeutic targets. However, the antitumor effects mediated by PVRL2, PVR, and other DNAM1 axis members remain to be elucidated and require further study.

## Author contributions

KM: Conceptualization, Writing – original draft. SG: Conceptualization, Writing – review & editing.
